# Wearable-Measured Physical Activity Goal Adherence and Body Composition Change in a 12-Month mHealth Weight Loss Trial

**DOI:** 10.3390/s26103256

**Published:** 2026-05-21

**Authors:** Zhadyra Bizhanova, Lora E. Burke, Maria M. Brooks, Bonny Rockette-Wagner, Jacob K. Kariuki, Susan M. Sereika

**Affiliations:** 1 School of Nursing, University of Pittsburgh, 3500 Victoria Building, Pittsburgh, PA 15261, USA; 2 School of Public Health, University of Pittsburgh, 130 De Soto St, Pittsburgh, PA 15261, USA; 3 Nell Hodgson Woodruff School of Nursing, Emory University, Atlanta, GA 30322, USA

**Keywords:** physical activity adherence, moderate-to-vigorous physical activity (MVPA), mobile health (mHealth), wearable activity trackers, wearable sensors, body composition, body fat, waist circumference, randomized controlled trial, weight loss, obesity, adults

## Abstract

**Highlights:**

**What are the main findings?**

- Higher adherence to wearable-measured physical activity goals was associated with greater reductions in percentage body fat and waist circumference among adults with overweight or obesity over 12 months.
- Associations between adherence to physical activity goals and percentage body fat and waist circumference differed by sex and treatment over time, with treatment-by-time interactions indicating smaller reductions in the intervention group, particularly among men.

**What are the implications of the main findings?**

- Sustained adherence to physical activity goals was more strongly associated with improvements in adiposity than intervention assignment alone, underscoring the central role of behavioral adherence in long-term weight management.
- Wearable activity trackers provide scalable, objective assessment of physical activity adherence, supporting their use for generating guideline-based evidence in long-term mobile health behavioral interventions.

**Abstract:**

Background: Wearable activity trackers are commonly used in mHealth weight loss interventions, but evidence linking adherence to moderate-to-vigorous physical activity (MVPA) goals with changes in body composition is limited. We examined associations between adherence to study-prescribed MVPA goals and changes in percent body fat and sex-specific waist circumference (WC) over 12 months in the SMARTER trial. Methods: Participants (N = 502, 79.5% female; mean age 45 years; mean BMI 33.7 kg/m^2^) were randomized to self-monitoring of diet, PA, and weight (SM) or SM plus daily tailored feedback messages (SM + FB). Weekly adherence to ≥300 min/week of MVPA was quantified using Fitbit-derived equivalents. Associations between MVPA adherence and changes in percent body fat and sex-specific WC over 12 months were examined using linear mixed models. Results: Among the full sample, greater MVPA adherence was associated with reductions in body fat (b = −0.01; 95% CI: −0.02, −0.005), but not in WC (women: b = −0.01; −0.03, 0.01; men: b = −0.03; −0.05, 0.0002). Among the completers, higher adherence was associated with decreases in body fat (b = −0.01; −0.02, −0.004) and WC (women: b = −0.02; −0.04, −0.004; men: b = −0.04; −0.08, −0.003). Conclusions: Higher MVPA adherence was associated with favorable changes in adiposity over 12 months, supporting the use of wearable-derived PA measures in long-term mHealth behavioral interventions.

## 1. Introduction

In 2022, approximately 2.5 billion adults worldwide were overweight, including more than 890 million living with obesity [1]. In the United States, obesity affected about 40% of adults between 2021 and 2023, with similar prevalence by sex, although severe obesity was more common in women than men (12.1% vs. 6.7%) [2]. Beyond overall adiposity, central fat accumulation is a critical determinant of cardiometabolic risk. Each 10 cm increase in waist circumference (WC) is associated with a 45% higher prevalence of cardiovascular disease and an 8% increase in all-cause mortality [3]. To reduce this risk, current national physical activity (PA) guidelines recommend 150–300 min of moderate-intensity or 75–150 min of vigorous-intensity aerobic physical activity (MVPA) per week [4,5]. Supporting these recommendations, the PREDIMED-Plus trial demonstrated that an energy-reduced Mediterranean diet combined with PA significantly reduced body fat over 3 years in older adults with overweight/obesity [6]. Given the importance of meeting the recommended MVPA levels, recent research has increasingly focused on the use of wearable activity trackers to support PA engagement among adults with overweight/obesity.

Mobile health (mHealth) interventions using wearable activity trackers offer scalable, cost-effective approaches to promote adherence to PA guidelines through self-monitoring and personalized feedback. However, evidence of their effectiveness in improving body composition among adults with overweight/obesity remains mixed, with most studies focusing on weight and BMI rather than adiposity-specific outcomes, such as body fat and WC [7,8,9,10,11]. An umbrella review of 39 systematic reviews showed that activity trackers improved body composition (SMD 0.7–2.0) and PA (SMD 0.3–0.6) [7]. However, effects on percent body fat and WC were not consistently reported across included studies [7]. In the IDEA trial, adding a wearable device to a standard behavioral weight loss intervention did not provide additional benefits, as both groups achieved similar improvements in body composition, PA, and diet [8]. In contrast, a meta-analysis of 18 RCTs reported significant WC reductions (−5.22 cm) in adults with overweight or obesity, despite no significant overall weight change, suggesting that wearable interventions may differently affect adiposity measures [11]. These inconsistent findings, particularly the limited and heterogeneous evidence on percent body fat and WC, underscore the importance of examining adherence to PA goals, rather than device use alone, as a predictor of intervention effectiveness.

Although several wearable-supported interventions have demonstrated associations between PA and weight loss outcomes [7,8,9,10,11], the role of adherence to PA goals in improving adiposity-specific outcomes has only recently been examined [12,13,14]. In the SMARTER trial, greater adherence to self-monitoring and behavioral goals was associated with increased odds of achieving ≥5% weight loss [12]. Moreover, a dose–response meta-analysis of 116 RCTs found that each additional 30 min/week of aerobic MVPA was associated with reductions in body weight (−0.52 kg), WC (−0.56 cm), and percent body fat (−0.37%) [13]. Despite this emerging evidence [12,13,14], the extent to which sustained adherence to PA goals is specifically related to changes in adiposity measures, such as percent body fat and WC, in the context of wearable-supported behavioral interventions remains largely unexplored.

Given that tailored feedback on self-monitoring data has been shown to improve self-monitoring and PA goal adherence in behavioral interventions [15], the SMARTER trial, a 12-month RCT (N = 502) that compared self-monitoring alone (SM) with self-monitoring plus daily tailored feedback (SM + FB) among adults with overweight or obesity [16], provided well-suited data to examine this question. While the original SMARTER trial focused on weight loss as the primary outcome [16], it did not examine whether adherence to PA goals was associated with adiposity-specific changes. Therefore, the aim of this secondary analysis was to evaluate whether adherence to a study-defined MVPA goal was associated with changes in percent body fat and sex-specific WC over 12 months among adults with overweight and obesity participating in a mHealth weight loss trial. Clarifying these relationships is critical for optimizing mHealth weight loss interventions to improve body composition beyond weight loss alone.

## 2. Methods

Study design and participants: SMARTER was a two-arm, parallel-design, randomized weight loss trial conducted in southwestern Pennsylvania, USA [16]. The study was conducted in accordance with the principles of the Declaration of Helsinki and was approved by the Institutional Review Board of the University of Pittsburgh (protocol PRO17040453; approved April 2017). All participants provided written informed consent. Participants were randomly assigned to one of two intervention groups: (1) self-monitoring (SM; n = 251) of diet using the Fitbit app, PA using Fitbit Charge 2™ (San Francisco, CA, USA) trackers, and body weight using research-issued smart scales (Withings, Issy-les-Moulineaux, France); or (2) self-monitoring using the same devices plus tailored daily feedback messages, tailored based on the uploaded self-monitoring data (SM + FB; n = 251).

Randomization was performed at the beginning of the baseline 1:1 session using a computer-based program with equal allocation (1:1) and generated via minimization, an adaptive randomization method used to balance treatment groups according to sex and race. Allocation assignments were generated electronically and were not known to study staff prior to assignment. Due to the behavioral nature of the intervention, participants and study staff were not blinded to treatment assignment.

Adults aged 18 years or older with a body mass index (BMI) between 27 and 43 kg/m^2^ who used a smartphone with a data plan and completed a 5-day run-in online food diary during pre-trial period were eligible to participate. Exclusion criteria included having a medical condition requiring a supervised diet or assistance with PA; planning pregnancy during the study period; having a serious mental disorder (e.g., schizophrenia); self-reporting consuming ≥4 alcoholic drinks/day; participating in a concurrent weight loss program or taking a weight loss medication; having a history of bariatric surgery; or scoring >32 on the Eating Habits Checklist. The study protocol, including a full list of inclusion and exclusion criteria, has been published elsewhere [16].

SMARTER intervention: At baseline, participants in both groups attended a one-on-one counseling session with a master’s-level and highly experienced registered dietitian focused on behavioral weight loss strategies and were granted access to online educational content adapted from the Diabetes Prevention Program (DPP). All participants received a Fitbit tracker and a smart digital weight scale. Research staff assisted participants with downloading the Fitbit app, setting up user accounts, and learning to operate the monitoring devices.

Dietary prescription: Both intervention groups were assigned daily calorie goals based on baseline body weight (<200 lbs. vs. ≥200 lbs.) and sex (1200 or 1500 kcal/day for women; 1500 or 1800 kcal/day for men) [16]. Dietary fat intake was prescribed at 25% of total daily caloric intake. Calorie goals could be individualized over the course of the intervention to support continued weight loss among participants who struggled to meet prescribed targets or to facilitate weight loss maintenance. Participants were instructed to record daily dietary intake using the Fitbit app, which permitted self-monitoring of food intake but did not provide extensive automated feedback to users. Through investigator access to the Fitbit database, study staff were able to monitor participants’ total caloric intake and fat grams based on daily calorie goals. No specific protein or carbohydrate intake targets were prescribed.

PA prescription: Participants in both groups were prescribed non-structured, non-supervised MVPA and were provided with a wrist-worn Fitbit Charge 2™ tracker to self-monitor PA, which synchronized with their smartphone. In adults with overweight or obesity, concurrent wear of the Fitbit Charge 2™ and ActiGraph GT3X+ showed substantial agreement for step counts (ICC = 0.93), but systematic overestimation of MVPA by 15 min/day and underestimation of light-intensity PA by 32 min/day [17]. Participants were instructed to increase PA gradually, primarily through walking, with an initial goal of ≥150 min/week of MVPA by week 12. Participants were encouraged to increase activity by 10 min/week, targeting ≥300 min/week by week 42 and maintaining this level through week 52 [16]. This prescription aligns with the 2018 U.S. Physical Activity Guidelines, which recommends ≥300 min/week of MVPA for long-term weight loss and weight maintenance [18]. All activities (e.g., walking, bicycling) counted toward MVPA goals, and participants were encouraged to accumulate PA throughout the day. Resistance training was not targeted as a part of this intervention. Both groups received in-person PA counseling at baseline. However, only participants in the SM + FB group received tailored PA-related feedback messages every other day (i.e., 3–4 times/week) as part of the intervention for the 12-month follow-up period.

Self-weighing: Participants in both intervention groups were instructed to weigh themselves at least once daily at home using the study-issued smart digital scale, wearing light clothing and no shoes.

Tailored feedback messages: In the SM + FB group, a server-based feedback algorithm used real-time self-monitoring data to deliver up to 3 tailored feedback messages per day during waking hours throughout the 12-month intervention. Each message addressed a single behavior, including dietary intake, PA, or weekly weight change. Messages were personalized based on participants’ self-monitoring data and progress toward behavioral goals. For example, dietary feedback addressed calorie and fat gram intake relative to daily targets (e.g., “Calorie intake is above your goal, while fat grams are right on target. Take a moment to start to plan ahead for tomorrow!”), PA feedback encouraged participants to reinforced MVPA levels (e.g., Your Fitbit is reporting more than 150 min of physical activity!”, and weight-related feedback provided strategies during plateaus (e.g., “Plateaus can happen. Take a look at your food diary and see if there are any changes you can make”).

Self-monitoring data were uploaded to the study-developed Awesome Data Acquisition Method (ADAM), which used automated monitoring to flag ≥7 consecutive days without diet, weight, or PA or drastic weight changes (>5 lbs.) [19]. The SMARTER app delivered up to three randomly timed daily prompts (morning, afternoon, evening) [16]. Prompts opened within one hour of receipt displayed a personalized feedback message based on the uploaded tracked data and were recorded as opened; otherwise, no feedback was shown and the prompt was recorded as missed. SM + FB participants could not pause, delay, or disable message delivery.

Body composition and anthropometric measures: Percent body fat and WC were assessed at baseline, 6 months, and 12 months by trained research staff using a Tanita body composition analyzer (Model BF-350, Tanita Corporation, Tokyo, Japan) and a Gulick II measuring tape, respectively [16]. Tanita devices are commonly used to estimate body fat percentage in population-based studies [20]. In March 2020, in-person assessments were suspended because of the COVID-19 pandemic, and participants could have missing percent body fat and WC measurements at 6 months, 12 months, or both due to transition to remote visits (see the details of the missingness patterns in the statistical analysis section). Analyses were conducted using all available data in the full sample and were compared with complete-case analyses including participants with measurements at all three time points to assess robustness of the findings. Primary outcomes were changes in percent body fat and sex-specific WC from baseline to 6 and 12 months, with negative values indicating reductions.

Adherence to MVPA goal: PA was summarized over the first 6 and 12 months using Fitbit-derived MVPA equivalents, defined as fairly active (3–6 metabolic equivalents of task [METs]) and very active (>6 METs) minutes [21]. PA data were considered valid if participants recorded ≥500 steps/day on at least 4 days per week. This threshold was selected to confirm device wear rather than activity and to distinguish non-wear from sedentary behavior.

Weekly MVPA was calculated by summing fairly active and very active minutes, averaging across valid days, and multiplying by seven. The primary explanatory variable was percent adherence to the study-defined MVPA goal, calculated as weekly MVPA divided by 300 min, multiplied by 100%, and averaged over the 6- and 12-month periods. Adherence could exceed 100%.

Psychosocial measures: Self-efficacy at baseline was assessed using the Self-Efficacy and Exercise Habits Survey, an 11-item scale measuring confidence in one’s ability to plan and execute activities to meet PA goals, including two subscales: sticking to exercise and making time for exercise, with higher scores indicating greater exercise self-efficacy [16]. Depressive symptoms at baseline were assessed using the Center for Epidemiological Studies Depression (CES-D) scale (range 0–46), with higher scores indicating greater symptoms of depression severity [16]. Mental health problems were assessed using a 43-item medical history form administered during Phase II screening [16]. Participants self-reported the presence or absence of mental health conditions (e.g., anxiety, depression), which was coded as a binary variable (yes vs. no).

Statistical analysis: Continuous variables were summarized as mean ± standard deviation (SD) for normally distributed data and as median (1st quartile, 3rd quartile) for non-normally distributed data. Categorical variables were reported as n (%). Between-group comparisons were performed using a pooled or separate-variance independent group *t*-test or Wilcoxon rank-sum test for continuous variables and the Ꭓ^2^ test for independence or Fisher’s exact test for categorical variables, as appropriate. Statistical significance was defined as *p* < 0.05. All analyses were conducted using SAS version 9.4 (SAS Institute Inc., Cary, NC, USA).

Regarding missing data patterns, follow-up missingness in adiposity outcomes was primarily attributable to the transition to remote visits during the COVID-19 pandemic. Complete body fat percentage data across all three time points were available for 180 participants (35.9%). Of the remaining participants, 5 (1.0%) were missing 6-month data, 156 (31.1%) were missing 12-month data, and 159 (31.7%) were missing both follow-up measurements. Baseline data were unavailable for 2 participants (0.4%) due to technical issues. Complete WC data were available for 146 females (36.6%) and 37 males (35.9%). Among females, 3 (0.8%) were missing 6-month data, 125 (31.3%) were missing 12-month data, and 125 (31.3%) were missing both follow-up measurements. Among males, 32 (31.1%) were missing 12-month data and 34 (33.0%) were missing both 6- and 12-month measurements. To maximize statistical power, primary analyses employed full-information maximum likelihood estimation using all available data, while complete-case analyses were restricted to participants with complete data at all time points during pre-pandemic period. Comparisons between participants completing an in-person visit (n = 180) and those with a remote visit or dropout (n = 322) demonstrated no significant differences except in age and prevalence of mental health problems (Table 1).

Associations between changes in body fat percentage and sex-stratified WC with MVPA goal adherence were examined using linear mixed-effects modeling over 6 and 12 months. MVPA goal adherence was included as the primary independent variable, with fixed effects for treatment group assignment, time, and the treatment group-by-time interaction. Primary analyses employed full-information maximum likelihood estimation using all available data. In the full sample (Table 2), the adjusted model for change in body fat included treatment, time, their interaction, self-efficacy for exercise habits (“Sticking to It” subscale score), and percentage of days adherent to calorie goals.

For females, the adjusted model for the change in WC included treatment, time, their interaction, self-efficacy for exercise habits (“Sticking to It” subscale score), and a binary variable for follow-up during the COVID-19 pandemic. For males, the adjusted model for the change in WC included treatment, time, their interaction, and the percentage of days adherent to calorie goals. In the complete-case analysis restricted to study completers with in-person visits during the pre-pandemic period (Table 3), only statistically significant variables were retained in the final parsimonious adjusted model.

Model assumptions were assessed through residual analysis, evaluation of influential observations, and overall model fit. The robustness of regression results was examined by comparing results from the full analytic sample with complete-case analyses.

## 3. Results

Of the 502 participants (see Table 1), most were female (79.5%) and White (82.5%), with a mean age of 45.0 ± 14.4 years and a mean BMI of 33.7 ± 4.0 kg/m^2^. At baseline, mean percentage body fat was 41.4 ± 6.8%, and mean WC was 105.0 ± 11.6 cm in females and 112.5 ± 11.4 cm in males. During the first week, median MVPA was 215.0 min/week (Q1–Q3: 119.0–346.0); 65.9% of participants achieved ≥150 min/week by week 1 and 32.1% achieved ≥300 min/week, with a median adherence to the ≥300 min/week MVPA goal of 71.7% (Q1–Q3: 39.7–115.3).

Participants completing an in-person visit (n = 180) were older than those with a remote visit or dropout (n = 322) (mean age: 47.4 ± 13.9 vs. 43.7 ± 14.5; *p* = 0.005). Mental health problems were less common in the in-person group (18.9% vs. 27.3%; *p* = 0.03). No other significant differences were observed between groups, including sex, education, employment, partner status, baseline self-efficacy for exercise habits (“Sticking to It” subscale score), cardiometabolic measures, percentage of days adherent to calorie goals or percentage of MVPA goal adherence at 12 months.

In parsimonious adjusted linear mixed models (see Table 2), higher MVPA goal adherence was associated with greater reductions in percent body fat (b = −0.01; 95% CI: −0.02, −0.005; *p* = 0.0006), with no significant difference between 6 and 12 months (*p* = 0.16). The main effect of treatment group was not significant (SM + FB vs. SM: b = 0.33, 95% CI: −0.32, 0.99; *p* = 0.047); however, a significant treatment-by-time interaction indicated smaller body fat reductions in the SM + FB group compared with the SM group over time (b = 0.64; 95% CI: 0.04, 1.24; *p* = 0.037). Higher self-efficacy for exercise habits (“Sticking to It” subscale score: b = −0.63; 95% CI: −0.90, −0.35; *p* < 0.0001) and greater percentage of days adherent to calorie goals (b = −0.04; 95% CI: −0.05, −0.02; *p* = 0.0002) were also significantly associated with body fat reductions.

For female WC, MVPA goal adherence was not associated with change in WC (b = −0.01; 95% CI: −0.03, 0.01; *p* = 0.23), although reductions were greater at 0.12 months than at 6 months (b = −0.80; 95% CI: −1.90, 0.30; *p* = 0.02). No significant treatment or treatment-by-time interactions were observed. Follow-up during the COVID-19 pandemic was associated with smaller WC reductions (b = 4.05; 95% CI: 2.57, 5.53; *p* < 0.0001), while self-efficacy for exercise habits (“Sticking to It” subscale score) was associated with greater reductions (b = −0.71; 95% CI: −1.36, −0.06; *p* = 0.03).

For male WC, higher MVPA goal adherence was not significantly associated with change in WC (b = −0.03; −0.05, 0.0002; *p* = 0.05), with no difference between 6 and 12 months (*p* = 0.99). A significant treatment-by-time interaction indicated smaller WC reductions over time in the SM + FB group compared with the SM group (b = 3.28; 95% CI: 0.24, 6.31; *p* = 0.04). Greater percentage of days adherent to calorie goals was associated with WC reductions (b = −0.09; 95% CI: −0.16, −0.01; *p* = 0.03).

In parsimonious adjusted linear mixed models restricted to study completers with in-person visits during the pre-pandemic period (see Table 3), the associations between MVPA goal adherence and changes in body fat were similar to those observed in the full sample. However, unlike the full sample, MVPA goal adherence was significantly associated with WC reductions in both sexes (women: b = −0.02; 95% CI: −0.04, −0.004; *p* = 0.02; men: b = −0.04; 95% CI: −0.08, −0.003; *p* = 0.04). Self-efficacy for exercise habits (“Sticking to It” subscale score), percentage of days adherent to calorie goals, and follow-up during the pandemic were not significantly associated with WC changes in this subsample.

## 4. Discussions

The aim of this secondary analysis of the SMARTER trial was to evaluate whether adherence to a study-defined MVPA goal was associated with changes in percent body fat and sex-specific WC over 12 months among adults with overweight or obesity. In the full sample, higher MVPA goal adherence was associated with greater reductions in percent body fat, with comparable effects at 6 and 12 months. A significant treatment group-by-time interaction indicated smaller reductions in body fat and male WC in the SM + FB group compared with the SM group. Higher self-efficacy and greater adherence to calorie goals were also associated with body fat reductions. Among women, follow-up during the COVID-19 pandemic was associated with attenuated WC changes, while higher self-efficacy was associated with greater WC decreases. Among men, greater calorie goal adherence was associated with greater WC decreases. In the complete-case analysis restricted to study completers with in-person visits during the pre-pandemic period, MVPA goal adherence was significantly associated with reductions in both body fat and WC in both sexes, while self-efficacy, calorie goal adherence, and pandemic follow-up were not. Overall, these findings highlight the importance of sustained MVPA goal adherence for improving adiposity outcomes in wearable-supported behavioral interventions.

The observed association between higher MVPA goal adherence and reductions in percent body fat is consistent with prior evidence suggesting that adiposity measures may be more sensitive than body weight to changes in PA [11,13,22]. In a target trial emulation among postmenopausal women, Li et al. reported gradual reductions in total and abdominal adiposity with increasing MVPA goal adherence over three years [22]. Similarly, the dose–response meta-analysis by Jayedi et al. demonstrated linear decreases in body fat percentage, WC, and visceral adipose tissue with each additional 30 min/week of aerobic MVPA, despite modest weight loss [13]. These findings, together with our results, suggest that sustained aerobic MVPA adherence may promote favorable changes in body composition through increased energy expenditure and reductions in adipose tissue, even in the absence of substantial weight loss. For example, a meta-analysis of long-term (≥6 months) 17 RCTs demonstrated that aerobic exercise produced significant visceral fat reductions (SMD −0.54), even in the absence of caloric restriction, underscoring the role of aerobic exercise in targeting central adiposity [23]. Our findings highlight the value of using wearable trackers to self-monitor MVPA adherence as a meaningful behavioral target in weight management interventions.

Associations between MVPA goal adherence and WC were sex-specific and varied by treatment group assignment. In the full sample, MVPA goal adherence was not significantly associated with WC in either sex. However, in the complete-case analysis restricted to pre-pandemic in-person visits, significant associations were observed for both sexes. This discrepancy may reflect the confounding influence of pandemic-related disruptions on WC measurement in the full sample, which attenuated the ability to detect the associations between MVPA goal adherence and WC changes. This finding also suggests that WC may serve as a distinct measure of adiposity change that responds differently to MVPA goal adherence than percent body fat. Prior evidence indicates that WC trajectories are shaped by sex and age-specific patterns that are not fully captured by BMI [24,25], and that increases in WC are more pronounced in women than men, suggesting that sex-specific physiological mechanisms independently influence central adiposity [24]. These findings underscore the importance of evaluating central adiposity separately by sex when examining WC in wearable-supported behavioral interventions.

Although no main effect treatment effect was observed, a significant treatment-by-time interaction indicated that reductions in adiposity were smaller over time in the SM + FB group compared with the SM group, particularly among men. This finding is consistent with prior evidence, including the IDEA randomized trial, which demonstrated no added benefit of wearable-supported interventions beyond standard behavioral approaches for weight outcomes [8]. In the present study, all participants received Fitbit feedback, including reward badges and weekly activity summaries. In the SM + FB group, participants additionally received remotely delivered, tailored feedback based on self-monitoring data. However, this required consistent adherence to self-monitoring of PA, weight, and diet, as well as regular device syncing with the Fitbit app to trigger feedback delivery, potentially introducing additional participant burden and limiting intervention effectiveness. These findings suggest that remotely delivered, tailored feedback alone may be insufficient to improve MVPA-related adiposity outcomes beyond standard wearable features. For example, a meta-analysis of 75 studies found that human counseling is the only intervention component with a significant moderating effect on PA, with phone or video-based counseling demonstrating the greatest impact [26], which suggests the importance of incorporating a human counseling component when optimizing intervention modalities to support MVPA adherence.

Despite significant associations between MVPA goal adherence and percentage body fat, fewer than one-third of our study’s participants achieved the ≥300 min/week MVPA goal, thus reducing the dose of the prescribed intervention and its potential effect on adiposity. These findings align with prior evidence that indicates that higher MVPA volumes and strategies supporting sustained adherence are critical for improving body composition in adults with overweight and obesity [18,27].

The observed associations of exercise self-efficacy and calorie goal adherence with body fat reductions in the present study align with prior studies [28,29]. Increases in self-efficacy have been shown to predict greater MVPA adherence and weight loss at 12 months [28]. Additionally, dietary adherence has been identified as critical for both short- and long-term weight loss [29]. These findings underscore the importance of self-efficacy and calorie goal adherence as modifiable determinants of body composition improvements in mHealth behavioral interventions.

This study has several strengths. Strengths include a large, randomized controlled trial and the use of objective, daily MVPA data collected over 12 months using commercial wearable trackers. Evaluating adherence to guideline-based MVPA goals facilitated interpretation and translation of findings for both research and policy contexts. Additionally, this study adds to the limited long-term evidence linking MVPA goal adherence with changes in body composition in adults with overweight and obesity.

Several limitations should be acknowledged. Body composition data were missing more frequently than anticipated due to COVID-19-related restrictions on in-person assessments, which may have reduced statistical power to detect clinically meaningful effects. Beyond missing data, pandemic-related restrictions may have influenced participants’ overall activity levels and health behaviors, as prior evidence has documented significant reductions in PA and increases in sedentary behavior during COVID-19 lockdowns [30], which may have attenuated intervention effects on adiposity outcomes. Additionally, first-week MVPA was used as a proxy for baseline activity and may have overestimated habitual PA due to monitoring awareness, although a brief run-in period would likely have mitigated this effect. Moreover, MVPA estimates were derived from Fitbit Charge 2™, which has been shown to systematically overestimate MVPA and underestimate light-intensity activity in adults with overweight or obesity [17], potentially introducing measurement error. These device-related accuracies, combined with first-week differences in PA levels [31], may have contributed to the large variability observed in MVPA across the sample, thereby reducing statistical power to detect meaningful between-group differences. Although participants received dietary guidance, including prescribed daily caloric goals and fat intake based on daily calorie goals [16], a comprehensive assessment of overall energy balance and macronutrient distribution was limited, which may have introduced variability in dietary composition and influenced observed changes in body composition. Finally, the predominantly female and White study population limits generalizability. These limitations should be considered when interpreting the findings.

Future research needs to focus on intervention strategies that promote sustained adherence to MVPA goals and translate this adherence into clinically meaningful improvements in adiposity in the context of mHealth behavioral weight loss programs. Given the limited efficacy of remotely delivered, tailored feedback observed in this study, hybrid models that integrate human counseling warrant further evaluation. Although contemporary wearable technologies have substantially reduced the burden of self-monitoring, participant engagement with these tools tends to decline over time [8]. Enhancements to digital platforms that support sustained daily use, combined with more personalized, behaviorally focused feedback from interventionists, may strengthen self-monitoring, goal adherence, and the overall effectiveness of wearable-supported interventions. Importantly, future studies need to prioritize the recruitment of racially, ethnically, and sex-diverse samples to improve generalizability.

## 5. Conclusions

In this secondary analysis of the SMARTER trial, greater adherence to the study-defined MVPA goal, assessed using commercial wearable trackers, was associated with larger reductions in percent body fat in the full sample and in WC among study completers with in-person visits during the pre-pandemic period over 12 months among adults with overweight or obesity. However, this association should not be interpreted as causal due to the secondary analysis nature of this study. Treatment-by-time interactions indicated smaller reductions in body fat and male WC over time in the SM + FB group compared with the SM group. Higher self-efficacy and greater adherence to calorie goals were also associated with body fat reductions. Among women, follow-up during the COVID-19 pandemic was associated with attenuated WC changes. Overall, these findings support the use of tracker-based PA assessment to estimate MVPA adherence and inform guideline-relevant evidence in long-term, wearable-supported behavioral interventions. Future research needs to include hybrid models integrating human counseling with enhanced digital platforms to promote sustained engagement and goal adherence and prioritize the recruitment of racially, ethnically, and sex-diverse samples to improve generalizability.

## Figures and Tables

**Table 1:** Baseline characteristics of SMARTER participants by follow-up visit status.

	Pre-Pandemic In-Person Visit *n* = 180	Remote Visit/Dropout *n* = 322 (205/117)	*p*
SM + FB; *n* (%)	88 (48.9)	163 (50.6)	0.71
Age, years; mean ± SD	47.4 ± 13.9	43.7 ± 14.5	0.005
Female; *n* (%)	144 (80)	255 (79.2)	0.83
Education, years; mean ± SD	16.5 ± 2.8	16.4 ± 2.8	0.53
Employed; *n* (%)	149 (82.8)	263 (81.7)	0.76
Partnered; *n* (%)	123 (68.3)	206 (64)	0.32
Hypertension; *n* (%)	40 (22.2%)	66 (20.5%)	0.65
Mental health problems; *n* (%)	34 (18.9%)	88 (27.3%)	0.03
Baseline self-efficacy for exercise habits (“Sticking to It” subscale score); mean ± SD	3.1 ± 1.0	3.0 ± 1.0	0.21
BMI, kg/m^2^; mean ± SD	33.4 ± 3.9	33.9 ± 4.0	0.15
Body fat, %; mean ± SD	41.1 ± 6.9	41.5 ± 6.7	0.53
Female WC, cm; mean ± SD	105.1 ± 10.2	105.5 ± 11.6	0.74
Male WC, cm; mean ± SD	112.2 ± 11.9	113.6 ± 12.4	0.59
MVPA goal adherence at 12 months, %; mean ± SD	85.8 ± 51.9	77.5 ± 50.3	0.08
Days adherent to calorie goals at 12 months, %; mean ± SD	43 ± 16.3	42.5 ± 18	0.76

Notes: cm, centimeters; MVPA, moderate- to vigorous-intensity physical activity; SD, standard deviation; SM + FB, Self-monitoring and Feedback; WC, waist circumference. Data are presented as mean (±SD) for continuous variables and n (%) for categorical variables. Group differences were assessed using parametric two-sample *t*-tests for continuous variables and chi-square tests for independence for categorical variables.

**Table 2:** Associations of MVPA Adherence with changes in percent body fat and sex-specific waist circumference (full sample).

	Change in Body Fat, % *n* = 500			Change in Female WC, cm *n* = 399			Change in Male WC, cm *n* = 103		
	Estimate 95% CI	*t*-Test *p*	F-Test *p*	Estimate 95% CI	*t*-Test *p*	F-Test *p*	Estimate 95% CI	*t*-Test *p*	F-Test *p*
MVPA Goal Adherence; %	−0.01 −0.02, −0.005	0.0006	0.0006	−0.01 −0.03, 0.01	0.23	0.23	−0.03 −0.05, 0.0002	0.05	0.05
Time Period; 0 to 12 months vs. 0 to 6 months (reference)	−0.10 −0.52, 0.32	0.64	0.16	−0.80 −1.90, 0.30	0.15	0.02	−1.63 −3.81, 0.55	0.14	0.99
Treatment Group; SM + FB vs. SM (reference)	0.33 −0.32, 0.99	0.32	0.047	−0.30 −1.74, 1.15	0.68	0.50	1.71 −0.36, 4.777	0.26	0.03
SM + FB × 0 to 12 months vs. SM × 0 to 6 months (reference)	0.64 0.04, 1.24	0.037	0.037	−0.36 −1.92, 1.20	0.65	0.65	3.28 0.24, 6.32	0.04	0.04
Baseline self-efficacy for exercise habits (“Sticking to It” subscale score)	−0.63 −0.90, −0.35	<0.0001	<0.0001	−0.71 −0.36, −0.06	0.03	0.03	----	----	----
% Days adherent to calorie goals	−0.04 −0.05, −0.02	0.0002	0.0002	----	----	----	−0.09 −0.16, −0.01	0.03	0.03
Follow-up during COVID-19 pandemic; Yes vs. No (reference)	----	----	----	4.05 2.57, 5.53	<0.0001	<0.0001	----	----	----

**Table 3:** Associations of MVPA adherence with changes in percent body fat and sex-specific waist circumference in study completers with in-person visit status during pre-pandemic period.

	Change in Body Fat, % *n* = 178			Change in Female WC, cm *n* = 144			Change in Male WC, cm *n* = 36		
	Estimate 95% CI	*t*-Test *p*	F-Test *p*	Estimate 95% CI	*t*-Test *p*	F-Test *p*	Estimate 95% CI	*t*-Test *p*	F-Test *p*
MVPA Goal Adherence; %	−0.01 −0.02, −0.004	0.005	0.005	−0.02 −0.04, −0.004	0.02	0.02	−0.04 −0.08, −0.003	0.04	0.04
Time Period; 0 to 12 months vs. 0 to 6 months (reference)	−0.04 −0.48, 0.40	0.86	0.10	−0.63 −1.77, 0.50	0.27	0.04	−1.52 −3.91, 0.86	0.20	0.92
Treatment Group; SM + FB vs. SM (reference)	0.53 −0.45, 1.50	0.29	0.08	0.25 −1.74, 2.25	0.80	0.99	5.01 0.22, 9.81	0.04	0.006
SM + FB × 0 to 12 months vs. SM × 0 to 6 months (reference)	0.61 −0.02, 1.24	0.06	0.06	−0.48 −2.12, 1.15	0.56	0.56	2.89 −0.38, 6.17	0.08	0.08
Baseline self-efficacy for exercise habits (“Sticking to It” subscale score)	−0.60 −0.93, −0.26	0.0005	0.0005	----	----	----	----	----	----
% Days adherent to calorie goals	−0.04 −0.07, −0.01	0.003	0.003	----	----	----	----	----	----

Notes: cm, centimeters; CI, confidence intervals; MVPA, moderate- to vigorous-intensity physical activity; SM + FB, Self-monitoring and Feedback; WC, waist circumference. Reported values are model coefficients (b) with 95% confidence intervals estimated from linear mixed models. *p*-values for individual fixed effects were derived from *t*-tests and overall effects were assessed using F-tests.

## Data Availability

The original contributions presented in this study are included in the article. Further inquiries can be directed to the corresponding author.
